# Unraveling the gut microbiota’s role in obesity: key metabolites, microbial species, and therapeutic insights

**DOI:** 10.1128/jb.00479-24

**Published:** 2025-04-04

**Authors:** Majid Iqbal, Qian Yu, Jingqun Tang, Juanjuan Xiang

**Affiliations:** 1 Hunan Key Laboratory of Early Diagnosis and Precise Treatment of Lung Cancer, The Second Xiangya Hospital 70566, Changsha, Hunan, China; 2 Cancer Research Institute, School of Basic Medical Science, Central South University 614453, Changsha, Hunan, China; 3 NHC Key Laboratory of Carcinogenesis and the Key Laboratory of Carcinogenesis and Cancer Invasion of the Chinese Ministry of Education, Xiangya Hospital, Central South University 159374, Changsha, Hunan, China; 4 Department of Thoracic Surgery, The Second Xiangya Hospital, Central South University 620731, Changsha, Hunan, China; University of Virginia School of Medicine, Charlottesville, Virginia, USA

**Keywords:** obesity, gut microbiota, inflammation, lipopolysaccharides, adipose tissue

## Abstract

Obesity, characterized by excessive fat accumulation, stems from an imbalance between energy intake and expenditure, with the gut microbiota playing a crucial role. This review highlights how gut microbiota influences metabolic pathways, inflammation, and adipose tissue regulation in obesity. Specific bacteria and metabolites, such as lipopolysaccharides (LPS) and short-chain fatty acids (SCFAs), modulate gut permeability, inflammation, and energy harvest, impacting obesity development. Certain gut bacteria, including *Clostridium XIVb*, *Dorea* spp., *Enterobacter cloacae*, and *Collinsella aerofaciens*, promote obesity by increasing energy harvest, gut permeability, and inflammatory response through LPS translocation into the bloodstream. Conversely, beneficial bacteria like *Akkermansia muciniphila*, *Lactobacillus* spp., and *Bifidobacterium* spp. enhance gut barrier integrity, regulate SCFA production, and modulate fasting-induced adipose factor, which collectively support metabolic health by reducing fat storage and inflammation. Metabolites such as SCFAs (acetate, propionate, and butyrate) interact with G-protein coupled receptors to regulate lipid metabolism and promote the browning of white adipose tissue (WAT), thus enhancing thermogenesis and energy expenditure. However, LPS contributes to insulin resistance and fat accumulation, highlighting the dual roles of these microbial metabolites in both supporting and disrupting metabolic function. Therapeutic interventions targeting gut microbiota, such as promoting WAT browning and activating brown adipose tissue (BAT), hold promise for obesity management. However, personalized approaches are necessary due to individual microbiome variability. Further research is essential to translate these insights into microbiota-based clinical therapies.

## INTRODUCTION

Obesity, a complex and multifactorial metabolic syndrome, is characterized by an imbalance between energy intake and expenditure, leading to excessive fat accumulation. Traditionally associated with caloric intake and physical inactivity, obesity is now increasingly understood to be influenced by the gut microbiota—a diverse and dynamic microbial community within the gastrointestinal tract (1). Emerging evidence suggests that gut microbiota is associated with host metabolism through bioactive metabolites (Tables 1 and 2), which may influence key processes such as lipogenesis, insulin sensitivity, systemic inflammation, and neurohormonal signaling (2). Associations between microbial dysbiosis—imbalances in microbial composition—and obesity, along with related comorbidities, have provided novel insights into the potential pathophysiology of these conditions. However, these findings are based largely on correlational data, and further research, particularly experimental or longitudinal studies, is required to elucidate causal mechanisms.

**TABLE 1:** Literature review of obesity-related gut microbes and their metabolites^*a*^

Study model	Bacterial strains	Impact on obesity	Metabolites	Pathomechanism	Therapeutic implications	Ref
*In vitro* HT-29 cells	*Parabacteroides distasonis*	Reduced	SCFAs and secondary bile acids	AMPK pathway activation, TGR5 pathway, and FXR pathway modulation	Probiotic development, metabolite production, and pathway modulation (AMPK, TGR5, and FXR)	(3)
C57BL/6J mice	*Parabacteroides goldsteinii*	Reduced	SCFAs	AMPK pathway	Probiotic supplementation prebiotic enhancement metabolite therapy (SCFA production, AMPK activation)	(4)
ApoE-null mice	*Parabacteroides merdae*	Reduced	Catabolism of branched-chain amino acids (BCAAs)	mTORC1 Pathway inhibition and catabolism of BCAA	Probiotic supplementation targeted microbiota modulation gene therapy (porA gene)	(5)
C57BL/6J mice on a high-fat diet	*Clostridium butyricum*	Reduced	Metabolism of tryptophan and purine	Tryptophan metabolism alteration and purine metabolism modulation	Oral supplementation of *Clostridium butyricum*	(6)
Human subjects with varying BMI (obese versus lean)	Sequencing and analysis of the participants' gut microbiota	*Dorea formicigenerans*, *Dorea longicatena*, and *Collinsella aerofaciens* were promoting obesity	SCFAs (acetate, propionate, butyrate) and BCAAs	Butyrate production pathway (SCFA metabolism), BCAA catabolism pathway, and LPS-TLR4 signaling pathway	Modulating the gut microbiota composition through dietary interventions	(7)
Overweight and obese pregnant women	*Collinsella* genus	Promoted	Bile acids and cholesterol	Potentially influencing lipid metabolism	Increase dietary fiber intake	(8)
Post-gastric bypass patients	*Dorea longicatena*	Promoted	Indole-3-acetate	Tryptophan metabolism pathway	Reduce red meat intake	(9)
*Caenorhabditis elegans*	*Enterobacter cloacae*	Promoted	SCFAs and LPS	Lipogenesis and TLR4 signaling pathway	Use of probiotic *Lactobacillus pentosus* MJM60383	(10)
Animal model (MAFLD)	*Akkermansia muciniphila*	Reduced	l-aspartate	l-aspartate metabolic pathway	*Akkermansia muciniphila* supplementation	(11)
Beagles	*Akkermansia muciniphila*	Reduced	SCFAs such as butyrate	Activation of AMPK pathway	*Akkermansia muciniphila* supplementation	(12)
C57BL/6 mice	*Lactobacillus rhamnosus* LS-8 and *Lactobacillus crustorum* MN047	Reduced	SCFAs, lactate, and bile acids	AMPK pathway (for SCFAs) FXR pathway (for bile acids)	Administration of *Lactobacillus rhamnosus* LS-8 and *Lactobacillus crustorum* MN047	(13)
Rodent and human subjects	*Bifidobacterium longum*	Reduced	SCFAs, tryptophan, and bile acids	AMPK, tryptophan metabolism, and FXR pathways	Administration of *Bifidobacterium longum*	(14)
C57BL/6J mice	*Lactobacillus gasseri* LG-G12	Reduced	SCFAs, particularly butyrate	Activation of AMPK pathway	Administration of *Lactobacillus gasseri* LG-G12	(15)
C57BL/6J mice	*Bifidobacterium animalis* subsp. *lactis* MN-Gup	Reduced	SCFAs such as butyrate and acetate	Activation of AMPK pathway	Administration of *Bifidobacterium animalis* subsp. *lactis* MN-Gup	(16)
C57BL/6J	*Escherichia coli* Nissle 1917	Reduced	SCFAs, bile acids, and indole derivatives	AMPK, FXR, and tryptophan metabolism pathways	Administration of genetically engineered *Escherichia coli* Nissle 1917 (EcN-GM)	(17)

^*a*^ Chamarande et al. showed that *Parabacteroides distasonis* reduced obesity *in vitro* via SCFAs, bile acids, and AMPK, TGR5, FXR pathways (3). Wu et al. showed that *Parabacteroides goldsteinii* reduced obesity in mice by regulating SCFAs and activating the AMPK pathway (4). In ApoE-null mice, *Parabacteroides merdae* reduced obesity by inhibiting the mTORC1 pathway, promoting BCAA catabolism, and suggesting potential probiotic therapies (5). In C57BL/6J mice on a high-fat diet, *Clostridium butyricum* reduced obesity by modulating tryptophan and purine metabolism (6). In human subjects, *Dorea formicigenerans, Dorea longicatena,* and *Collinsella aerofaciens* promoted obesity through SCFA metabolism, BCAA catabolism, and LPS-TLR4 signaling (7). In overweight and obese pregnant women, *Collinsella genus* promoted obesity by influencing bile acids, cholesterol levels, and lipid metabolism (8). In post-gastric bypass patients, *Dorea longicatena* promotes obesity via indole-3-acetate production, recommending reduced red meat intake to improve metabolic outcomes (9). In *Caenorhabditis elegans, Enterobacter cloacae* promotes obesity through SCFAs and LPS, with *Lactobacillus pentosus MJM60383* as a potential therapeutic strategy (10). In an MAFLD animal model, *Akkermansia muciniphila* reduces the condition by modulating the l-aspartate metabolic pathway, suggesting supplementation as a therapeutic approach (11). In beagles, *Akkermansia muciniphila* reduces obesity by producing SCFAs like butyrate, activating the AMPK pathway, suggesting supplementation for metabolic health (12). In C57BL/6 mice, *Lactobacillus rhamnosus LS-8* and *Lactobacillus crustorum MN047* reduce obesity by producing SCFAs, lactate, and bile acids, activating the AMPK and FXR pathways, suggesting supplementation for metabolic health (13). In rodent and human subjects, *Bifidobacterium longum* reduces obesity by producing SCFAs, tryptophan, and bile acids, activating the AMPK and FXR pathways, suggesting supplementation for metabolic health (14). In C57BL/6J mice, *Lactobacillus gasseri* LG-G12 reduces obesity by producing SCFAs, particularly butyrate, activating the AMPK pathway, suggesting supplementation for metabolic health (15). In C57BL/6J mice, *Bifidobacterium animalis* subsp. *Lactis *MN-Gup reduces obesity by producing SCFAs like butyrate and acetate, activating the AMPK pathway, suggesting supplementation for metabolic health (16). In C57BL/6J mice, genetically engineered *Escherichia coli* Nissle 1917 (EcN-GM) reduces obesity by producing SCFAs, bile acids, and indole derivatives, activating the AMPK and FXR pathways, and modulating tryptophan metabolism, suggesting supplementation for metabolic health (17).

**TABLE 2:** Associated metabolites utilized by gut microbiota in promoting and reducing obesity

Metabolites	Associated microbiota	Pathomechanism	Role in obesity	Hypothetical treatment
PAMPs	*C. XIVb*, *D. formicigenerans*, *D. longicatena*, *C. aerofaciens*, *E. cloacae*, *L. rhamnosus*, *L. gasseri*, *A. muciniphila*, *B. longum*, *B. animalis*, *E. coli* Nissle 1917	Activation of TLR4, NF-κB, leaky gut, downregulation of FIAF, and inhibition of AMPK.	Context-dependent, with some promoting while others reduce it.	Modulation of gut microbiota, inhibition of TLR4 signaling, strengthening gut barrier integrity, regulation of FIAF, promotion of SCFA production, particularly butyrate.
SCFAs	*E. cloacae*, *C. aerofaciens*, *A. muciniphila*, *L. rhamnosus*, *L. gasseri*, *B. longum*, *B. animalis*, *E. coli*	Secretion of GLP-1 and PYY, activation of AMPK, and hepatic lipogenesis.	Context-dependent, dual role.	Gut microbiota modulation, promoting butyrate-producing bacteria, regulating SCFA metabolism, inhibiting obesogenic bacteria.
Bile acids	*E. cloacae*, *C. aerofaciens*, *A. muciniphila*, *L. rhamnosus*, *L. gasseri*, *B. longum*, *B. animalis*, *E. coli* Nissle 1917	Activation of FXR and TGR5, emulsification of lipid.	Context-dependent, dual role.	Nuclear receptor activation (FXR, TGR5), anti-obesogenic bacteria, inhibition of obesogenic bacteria, gut microbiota modulation.
Endocannabinoids	*E. cloacae*, *C. aerofaciens*, *A. muciniphila*, *L. rhamnosus*, *L. gasseri*, *B. longum*, *B. animalis*, *E. coli* (Nissle 1917	Activation of CB1 and CB2 receptors, and orexigenic signaling.	Context-dependent, dual role.	CB1 receptor antagonism, CB2 receptor activation, and gut microbiota modulation.
Oxylipins	*E. cloacae*, *C. aerofaciens*, *A. muciniphila*, *L. rhamnosus*, *L. gasseri, B. longum*, *B. animalis*	Pro-inflammatory and anti-inflammatoryoxylipin pathways.	Context-dependent, dual role.	Promote anti-inflammatory oxylipin production, inhibit pro-inflammatory oxylipin production, and enhance beneficial gut bacteria.
Succinate	*E. Cloacae, C. aerofaciens, A. muciniphila, L. rhamnosus, L. gasseri, B. longum, B. animalis, E. coli* Nissle 1917	SUCNR1 activation, glycolytic shift, and succinate conversion to propionate.	Context-dependent, dual role.	Inhibit pro-inflammatory succinate pathways and promote anti-obesity succinate metabolism.
FIAF	*E. cloacae*, *C. aerofaciens*, *A. muciniphila*, *L. rhamnosus*, *L. gasseri*, *B. longum*, *B. animalis*, *E. coli* Nissle 1917	FIAF inhibition of LPL, gut microbiota modulation of FIAF expression, dysbiosis, and FIAF expression.	Context-dependent, dual role.	Promote FIAF upregulation, inhibit LPL activity, and target bacteria that downregulate FIAF.

Currently, an estimated 2.6 billion people, or 40% of the global population, are affected by overweight or obesity, with projections suggesting that this could rise to over 4 billion people by 2035, or roughly half of the global population (research by the World Obesity Federation). The increasing prevalence of obesity reflects a complex interplay of diet, lifestyle, genetic, environmental, and behavioral factors. Adipose tissue, once considered merely a storage site for energy, is now recognized as an active endocrine organ involved in metabolic regulation (18, 19). It is categorized into white adipose tissue (WAT) and brown adipose tissue (BAT), each with distinct roles in energy metabolism. WAT, primarily located in subcutaneous and visceral depots, stores triglycerides. Subcutaneous WAT may offer protective metabolic effects, whereas visceral WAT is closely linked to metabolic dysfunction and increased cardiovascular risk (18, 20, 21). In contrast, BAT, primarily responsible for non-shivering thermogenesis, contributes to energy expenditure and metabolic health (22). Beige adipose tissue, an intermediary between WAT and BAT, possesses some thermogenic capacity, though its physiological relevance in humans remains under investigation (23).

In obesity, shifts in gut microbiota composition often include a higher Firmicutes-to-Bacteroidetes ratio, decreased *Bacteroides*, and increased *Lactobacillus* and *Clostridium*. This dysbiosis is associated with increased energy extraction, chronic low-grade inflammation, and disrupted lipid metabolism (24, 25). Obesogenic bacteria, such as *Clostridium XIVb*, *Dorea* spp., *Enterobacter cloacae*, and *Collinsella aerofaciens*, are linked to increased gut permeability and inflammation (26). Meanwhile, beneficial bacteria like *Akkermansia muciniphila*, *Lactobacillus rhamnosus*, and *Bifidobacterium longum* play crucial roles in supporting gut barrier integrity and metabolic health (27). Various studies have shown that different gut microbiota species have the potential to either reduce or promote obesity through various mechanisms. *Parabacteroides distasonis*, *Parabacteroides goldsteinii*, and *Parabacteroides merdae* have been shown to reduce obesity *in vitro* and in mice by regulating SCFAs, bile acids, AMP-activated protein kinase (AMPK), G protein-coupled bile acid receptor 1 (TGR5), farnesoid X receptor (FXR) pathways, and the mTORC1 pathway. On the other hand, *Dorea formicigenerans*, *Dorea longicatena*, *C. aerofaciens*, the *Collinsella* genus, and *E. cloacae* have been found to promote obesity through different mechanisms (Table 1). This review explores the intricate relationships between gut microbiota, WAT, and BAT, with particular emphasis on short-chain fatty acid (SCFA) production, modulation of fasting-induced adipose factor (FIAF), and immune response regulation. By understanding these interactions, we aim to highlight potential microbiota-targeted therapeutic strategies for obesity, stressing the need for personalized approaches to accommodate individual microbiome variability (Tables 2 and 3).

**TABLE 3:** Clinical trials

Trial ID and procedure	Phase	Sponsor; Collaborator	Official title	Outcome measure
NCT02970877 FMT	Phase 2	Johane Allard	Fecal Microbiota Transplant from Healthy Lean Donors to Morbidly Obese Individuals: Effect on Insulin Resistance and Other Obesity-related Parameters. A Randomized Controlled Trial.	Change in insulin resistance compared to baseline
NCT03391817 FMT	N/A	Joint Authority for Päijät-Häme Social and Health Care	Fecal Microbiota Transplantation in the Treatment of Morbid Obesity.	Reduction of weight
NCT03789461 FMT	N/A	Chinese University of Hong Kong	An Open-label Pilot Study of Fecal Microbiota Transplant (FMT) to Induce Weight Loss in Obese Subjects	Proportion of at least 10% reduction in weight
NCT04579263 FMT	N/A	Federal Research and Clinical Center of Physical-Chemical Medicine	Assessment of Improvement in Glycemic Control, Weight, and Insulin Sensitivity in Obese Patients After Fecal Microbiota Transplantation (FMT) Against the Background of Glucose-lowering Therapy	Change in insulin sensitivity within FMT, 6 months after FMT
NCT03273855 FMT	N/A	University Hospital of North Norway	Randomized Controlled Trial of Fecal Microbiota Transplantation in Severe Obesity	Change in individual weight loss
NCT02741518 FMT	Phase 1	Brigham and Women’s Hospital	Fecal Microbiota Transplantation for the Treatment of Obesity	Adverse event frequency
NCT06268990 FMT	N/A	Wiebke Kristin Fenske	Metabolic Outcome of Obese Subjects Receiving Fecal Microbiota Transplantation of Lean Versus Gastric Bypass Treated Subjects. A Pilot Study	Insulin sensitivity
NCT03127696 FMT	N/A	Chinese University of Hong Kong	A Randomised Placebo-controlled Study of Fecal Microbiota Transplant (FMT) to Impact Body Weight and Glycemic Control in Obese Subjects with Type 2 Diabetes Mellitus	FMT in promoting lean-associated microbiota
NCT02180191	N/A	Gulhane School of Medicine	Comparison of Gut Microbiota in Obese, Diabetic, and Healthy Control Individuals.	Gut microbiota composition

## GUT MICROBES AND PATHOGEN-ASSOCIATED MOLECULAR PATTERN (PAMPs) IN OBESITY

PAMPs, particularly lipopolysaccharides (LPS) from gram-negative bacteria, are naturally present in the gut under homeostatic conditions. These PAMPs, including LPS, interact with pattern recognition receptors like Toll-like receptor 4 (TLR4), which normally contribute to immune surveillance and homeostasis. However, when this balance is disrupted—such as during obesity—these interactions can lead to pathological immune responses. LPS binding to TLR4 activates immune responses through nuclear factor-kappa B (NF-κB), promoting the release of pro-inflammatory cytokines like tumor necrosis factor-alpha (TNF-α), IL-1β, and IL-6 (28–30). This chronic, low-grade inflammation, termed metabolic inflammation, disrupts insulin signaling in key tissues, resulting in systemic insulin resistance, a central feature of obesity and metabolic syndrome. Furthermore, LPS-induced inflammation is associated with impaired gut barrier integrity, or “leaky gut,” allowing microbial products to enter the bloodstream and further amplify inflammation (31). Various factors such as a high-fat diet, excessive alcohol intake, obesity, hyperglycemia, and low dietary fiber contribute to gut barrier dysfunction, enhancing the translocation of endotoxins like LPS, which trigger inflammation and perpetuate metabolic disruption (32–34). In adipose tissue, PAMPs exacerbate macrophage infiltration and cytokine production, impairing insulin sensitivity and promoting lipid accumulation (35). As highlighted in seminal work by the Jeff Gordon lab, an altered gut microbiota in obesity, characterized by an increased Firmicutes-to-Bacteroidetes ratio, can intensify inflammation and metabolic dysfunction (24, 36). Nonetheless, Firmicutes, being gram-positive, do not produce LPS, which is mainly produced by gram-negative bacteria. Therefore, a higher Firmicutes-to-Bacteroidetes ratio could theoretically reduce LPS levels. However, the relationship between gut microbiota and metabolic outcomes is complex, involving factors beyond LPS, such as microbial metabolites and immune modulation.

**Fig 1:**
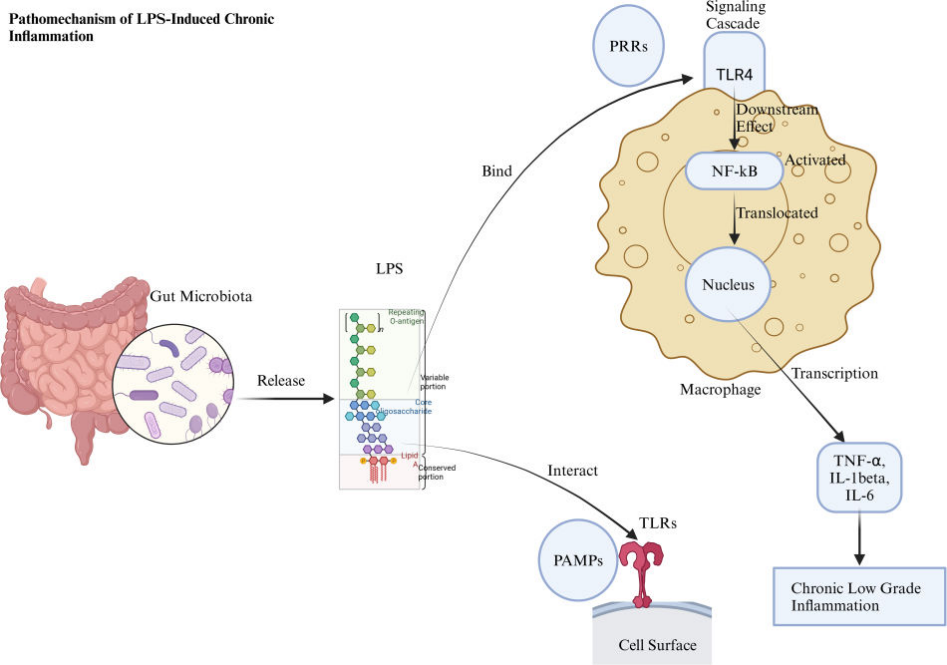
Lipopolysaccharides (LPS), released from gut microbiota, interact with Toll-like receptor 4 (TLR4) on macrophages, acting as a pathogen-associated molecular pattern (PAMP). Under homeostatic conditions, this binding plays a role in immune regulation, but when disrupted, it triggers an intracellular signaling cascade, activating nuclear factor-kappa B (NF-κB) as a downstream effect of TLR4 engagement. Once activated, NF-κB translocates to the nucleus and initiates the transcription of pro-inflammatory cytokines like TNF-α, IL-1β, and IL-6. This cytokine production contributes to chronic low-grade inflammation, leading to systemic insulin resistance, obesity, metabolic dysregulation, and a compromised gut barrier, commonly referred to as “leaky gut.” Created with BioRender.com.

LPS also interferes with adipocyte function by downregulating FIAF and inhibiting AMPK, promoting lipid synthesis and storage. Exposure to LPS affects adipogenesis by inhibiting transcription factors like peroxisome proliferator-activated receptor (PPAR) gamma and CCAAT/enhancer-binding protein alpha, essential for adipocyte differentiation, while inducing pro-inflammatory cytokines like TNF, which hinder adipogenesis through pathways such as wingless/integrated β-catenin T (WNT–β-catenin–T) cell factor 4 (TCF4) (37–40). Additionally, LPS alters adipokine levels, impacting apelin, adiponectin, and leptin, which regulate energy metabolism and inflammation (41, 42). Interestingly, LPS’s effects on adipose tissue and metabolism vary depending on concentration, duration, and context. For instance, LPS from *Escherichia coli* promotes inflammation and disrupts glucose metabolism, while LPS from *Rhodobacter sphaeroides* lacks these detrimental effects, suggesting that molecular variations, such as lipid A acylation, influence LPS’s impact on metabolic health (43). Beyond LPS, other PAMPs like peptidoglycans and lipopeptides contribute to metabolic disturbances. Peptidoglycans, found in both gram-positive and gram-negative bacteria, activate nucleotide-binding oligomerization domain containing 1 signaling, triggering lipolysis and inflammatory pathways (44). Other bacterial components like flagellin, DNA, and lipoproteins, which enter circulation due to increased gut permeability, are implicated in obesity and metabolic disorders (45–47). Certain PAMPs, such as LPS and flagellin, are found in both commensal and pathogenic bacteria. However, their immune outcomes can vary dramatically, ranging from immune tolerance to robust inflammation. One possible explanation lies in the molecular context in which these PAMPs are recognized by the host (48, 49). It is well established that the engagement of different immune receptors or co-receptors can lead to varying immune responses. For example, LPS from commensal bacteria may preferentially interact with receptors that promote immune tolerance, whereas LPS from pathogenic bacteria may engage different receptors or co-receptors, resulting in an inflammatory response (50). Additionally, the presence of host-microbe cross-talk and systemic signals could play a crucial role in modulating local immune responses. Factors such as tissue localization of bacteria, the presence of certain cytokines, or the activation of specific immune pathways could shape how PAMPs are perceived and how the immune system reacts (51, 52). While LPS is recognized as a microbial molecule that contributes to inflammatory pathways, particularly in obesity, the immune responses elicited by LPS can vary depending on its source, such as whether it originates from pathogenic or commensal bacteria. This dual role of LPS, which can either promote or mitigate inflammation, warrants further exploration. One potential explanation for these contrasting immune responses may lie in structural variations in the LPS molecules themselves. Research has shown that the lipid A component of LPS, which is responsible for its immunogenic activity, can vary in structure between different bacterial species, potentially influencing its ability to activate immune cells (50). Additionally, the association with specific bacterial species might contribute to differential immune outcomes. Commensal bacteria are typically more tolerogenic, and their LPS may interact with the host’s immune system in a way that promotes immune tolerance rather than activation. Moreover, the interaction between multiple microbial molecules may further influence the immune response. For instance, the presence of flagellin alongside LPS may create a synergistic effect, amplifying the inflammatory response. Alternatively, certain microbial metabolites or signaling molecules may modulate the recognition of PAMPs, altering the host’s immune response (53, 54). The diversity in bacterial PAMP effects may stem from differences in their interactions with the host’s immune system, metabolic pathways, and gut microenvironment. Certain beneficial bacteria, such as *A. muciniphila*, *L. rhamnosus*, and *B. longum*, produce similar PAMPs but promote immune homeostasis, strengthen gut barrier function, and support metabolic health (Table 2) (55, 56). Furthermore, bacteria that release anti-inflammatory metabolites like SCFAs may counteract the pro-inflammatory effects of PAMPs, promoting gut health and reducing obesity risk (Fig. 1). Microbial diversity within the gut microbiome may buffer against the obesity-promoting effects of PAMPs.

Host-specific factors, such as genetic predisposition, metabolic state, and environmental conditions, shape immune responses to PAMPs. Timing and duration of PAMP exposure influence whether responses are protective or harmful, reflecting the complex interplay between microbial components, host traits, and external factors (57). Tissue localization and immune priming may also modulate PAMP recognition. LPS in the gut may promote tolerance, while in the bloodstream, it may trigger robust inflammation (58). Additionally, the gut microbiota’s composition and its metabolic products, such as SCFAs, play a key role in determining immune responses. Commensal bacteria promote tolerance and gut health, whereas pathogenic bacteria or dysbiosis drive inflammation and metabolic dysfunction (50). External factors like diet, stress, and antibiotics may further impact these responses. High-fat diets promote inflammation, while fibers and polyphenols support anti-inflammatory microbes. Stress and antibiotic use disrupt the microbiota, worsening inflammation and metabolic diseases (59, 60). This dynamic interaction underscores the importance of understanding the context in which PAMPs are recognized. Targeting bacterial PAMPs and immune pathways may provide therapeutic strategies for obesity and related disorders. Future research should explore these mechanisms to clarify the microbiota’s role in disease.

## SCFAs AND KEY RECEPTORS

Humans lack the enzymes needed to digest dietary fibers, so these undigested carbohydrates pass through the upper gastrointestinal tract to the large intestine, where they are fermented by anaerobic gut bacteria. This fermentation generates SCFAs such as acetate, butyrate, and propionate (61–63). The amount and type of fiber consumed influence the gut microbiota composition, which directly affects SCFA production. SCFAs are vital for metabolic health, providing up to 10% of daily caloric intake and acting as a primary energy source for colonocytes, maintaining gut barrier integrity by reducing intestinal permeability (63). Colonocytes, the epithelial cells of the colon, primarily use SCFAs, especially butyrate, as an energy source. Butyrate is metabolized in mitochondria, producing adenosine triphosphate to support colonocyte functions like ion transport, mucus production, and maintaining the intestinal barrier (64). SCFAs, particularly butyrate, may also influence gene expression in colonocytes through epigenetic mechanisms, such as inhibiting histone deacetylases. This may result in histone acetylation, modulating genes involved in inflammation, cell proliferation, and apoptosis, crucial for gut homeostasis (65). Furthermore, SCFAs reduce intestinal inflammation by suppressing pro-inflammatory cytokines and promoting anti-inflammatory pathways. This anti-inflammatory effect can be key in preventing conditions like inflammatory bowel disease, highlighting the importance of SCFAs in regulating immune responses within the gut (66). By maintaining colonocyte function and reducing inflammation, SCFAs contribute to balanced host–microbe interactions, essential for overall health.

SCFA production is influenced by the gut microbiota, and these metabolites play a role in shaping the microbial ecosystem. SCFAs help lower gut pH, which encourages the growth of beneficial, SCFA-producing bacteria, such as *Faecalibacterium prausnitzii* and *Roseburia*, while inhibiting harmful bacteria like *Clostridium difficile* (67). These interactions promote a balanced microbiota and support gut health. Moreover, SCFAs may influence microbial communication through quorum sensing. By regulating signaling molecules, SCFAs can modulate microbial behaviors, including biofilm formation, virulence, and antimicrobial resistance (68). These changes in microbial interactions can influence the host’s metabolism and susceptibility to diseases, indirectly linking SCFAs to metabolic health.

SCFAs play a crucial role in regulating energy metabolism and influencing obesity development. SCFAs, especially acetate, propionate, and butyrate, promote the release of anorexigenic hormones like glucagon-like peptide 1 (GLP-1), peptide YY (PYY), and leptin. These hormones signal satiety, reduce food intake, and regulate energy expenditure (69). SCFAs activate G protein-coupled receptors (GPR41 and GPR43) on enteroendocrine cells, stimulating signaling pathways that influence lipid metabolism, glucose homeostasis, and insulin sensitivity (69, 70). In addition to regulating appetite, SCFAs also impact fat storage and adipocyte differentiation. SCFAs modulate fat metabolism and insulin sensitivity, and disturbances in SCFA production or absorption can lead to obesity and related metabolic disorders (71, 72). Dietary fiber or SCFA supplementation has shown promise in alleviating high-fat diet-induced obesity in animal models, suggesting that SCFAs could help restore metabolic balance and reduce the risk of obesity-related complications (69, 70).

**Fig 2:**
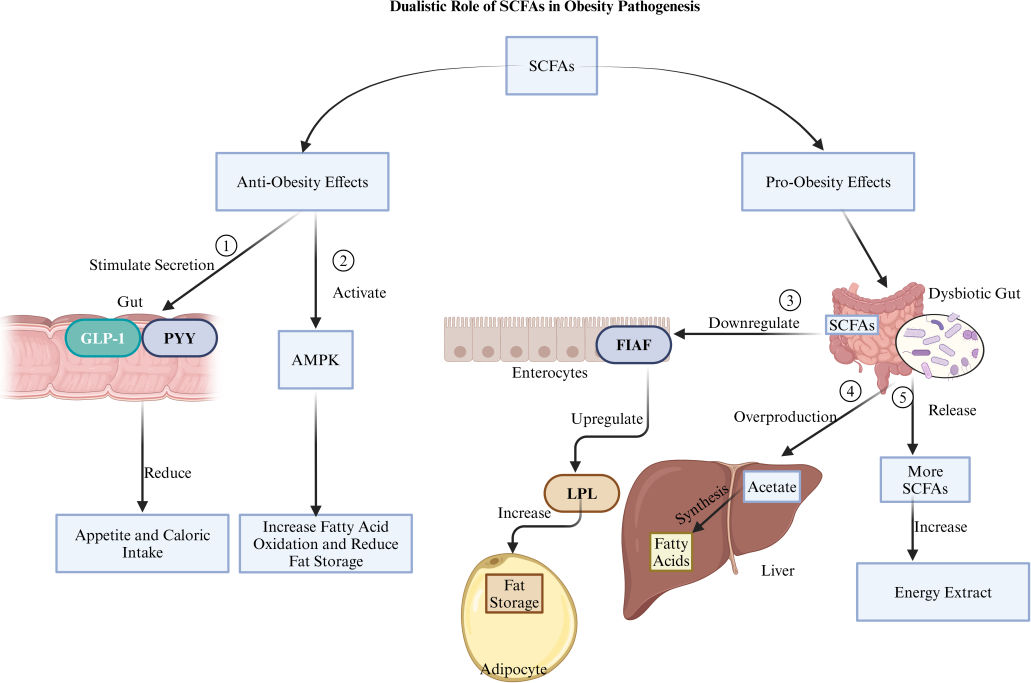
The dual role of SCFAs in obesity pathogenesis is as follows: (1) SCFAs show an anti-obesity effect by stimulating the secretion of GLP-1 and PYY in the gut, which reduces appetite and caloric intake. (2) They also exhibit an anti-obesity effect by activating the AMPK pathway, which increases fatty acid oxidation and reduces fat storage. (3) Conversely, SCFAs demonstrate a pro-obesity effect in a dysbiotic gut by downregulating FIAF in enterocytes, leading to the upregulation of LPL activity and increased fat storage in adipocytes. (4) Another pro-obesity effect arises from the overproduction of acetate due to dysbiosis, where this acetate is used by the liver to synthesize fatty acids, contributing to fat storage. (5) Finally, the figure highlights the pro-obesity effect of dysbiosis, which leads to the release of more SCFAs, resulting in increased energy extraction and fat storage. Created with BioRender.com.

However, the relationship between SCFAs and adipose tissue is complex. In certain contexts, SCFAs promote beneficial effects on metabolism, while in others, they may contribute to adiposity (Fig. 2). Butyrate can enhance adipogenesis through GPR43 activation, whereas propionate may stimulate lipogenesis in mature adipocytes via GPR41 (73). In adipose tissue, GPR41 and GPR43 activation can encourage adipocyte differentiation and hyperplasia, leading to increased fat mass. While SCFAs can promote adiposity under specific conditions, they also have the potential to favorably modulate metabolic processes. In BAT, acetate can upregulate genes and proteins linked to adipocyte differentiation, mitochondrial biogenesis, and thermogenesis. Mouse studies have shown that acetate enhances the expression of adipocyte protein 2 (AP2), PGC1α, and UCP1, increasing mitochondrial activity (74). However, these effects seem to differ between species, with more limited impact observed in human adipocytes. Butyrate stands out for its metabolic benefits, not only by improving gut health but also by modulating systemic energy metabolism. Studies in mice indicate that butyrate reduces food intake by acting on the gut-brain axis, promoting satiety, and inhibiting orexigenic neurons in the brain (75). Butyrate supplementation for an extended period in mice prevents diet-induced obesity and improves markers like insulin sensitivity and lipid profiles by enhancing fatty acid oxidation and increasing sympathetic outflow to BAT (75). These effects rely on the vagus nerve, as vagotomy abolishes butyrate’s impact on food intake and BAT activity. Additionally, SCFAs influence gene expression related to metabolism through epigenetic regulation, particularly by inhibiting histone deacetylases and affecting DNA methyl transferase activity (76). This modification of chromatin structure and DNA methylation alters gene expression, potentially supporting metabolic homeostasis and reducing the risk of metabolic disorders.

The dualistic nature of SCFAs is highlighted by their role in obesity pathogenesis, as their impact can shift based on metabolic and microbial context (Fig. 2). In a healthy gut, SCFAs help regulate appetite, energy balance, and metabolic health. However, in dysbiosis, SCFAs can exacerbate obesity by facilitating increased energy harvest, with acetate acting as a substrate for hepatic lipogenesis and promoting adipose tissue expansion (71). Certain bacteria, such as *E. cloacae* and *C. aerofaciens*, may drive obesity by enhancing SCFA production to increase energy harvest and hepatic lipogenesis (7, 26). Conversely, beneficial bacteria like *A. muciniphila*, *L. rhamnosus*, *Lactobacillus gasseri*, *B. longum*, and *Bifidobacterium animalis* support anti-obesity effects by producing SCFAs that strengthen gut barrier integrity, reduce inflammation, and enhance energy expenditure (12–15). Genetically modified *E. coli* Nissle 1917 (EcN-GM), a probiotic, may contribute to SCFA production, particularly acetate, which plays a role in metabolic balance (17).

## BILE ACIDS

Bile acids, primarily produced by the liver, play essential roles in both digestion and metabolic regulation, with their activities strongly influenced by the gut microbiota. The liver synthesizes primary bile acids, such as cholic acid and chenodeoxycholic acid (CDCA) in humans, conjugating them with glycine or taurine before storing them in the gallbladder (77–79). Upon food intake, bile acids are released into the small intestine to aid in fat digestion and absorption. Roughly 95% of these bile acids are reabsorbed in the ileum and recycled back to the liver, while the remaining fraction reaches the colon, where it can either be reabsorbed or excreted in feces (80). Beyond their role in fat digestion, bile acids act as signaling molecules, influencing glucose, lipid, and energy metabolism (78). Key receptors for bile acids, such as FXR and TGR5, mediate many of these effects (81). FXR activation in the liver and intestines inhibits hepatic lipogenesis, enhances insulin sensitivity, and increases energy expenditure, collectively reducing obesity risk. Additionally, bile acids help maintain gut barrier integrity, preventing LPS from entering the bloodstream and triggering systemic inflammation—a contributing factor in obesity and metabolic syndrome (82). Furthermore, bile acids support the growth of beneficial gut microbiota, fostering a favorable metabolic profile that reduces adiposity.

**Fig 3:**
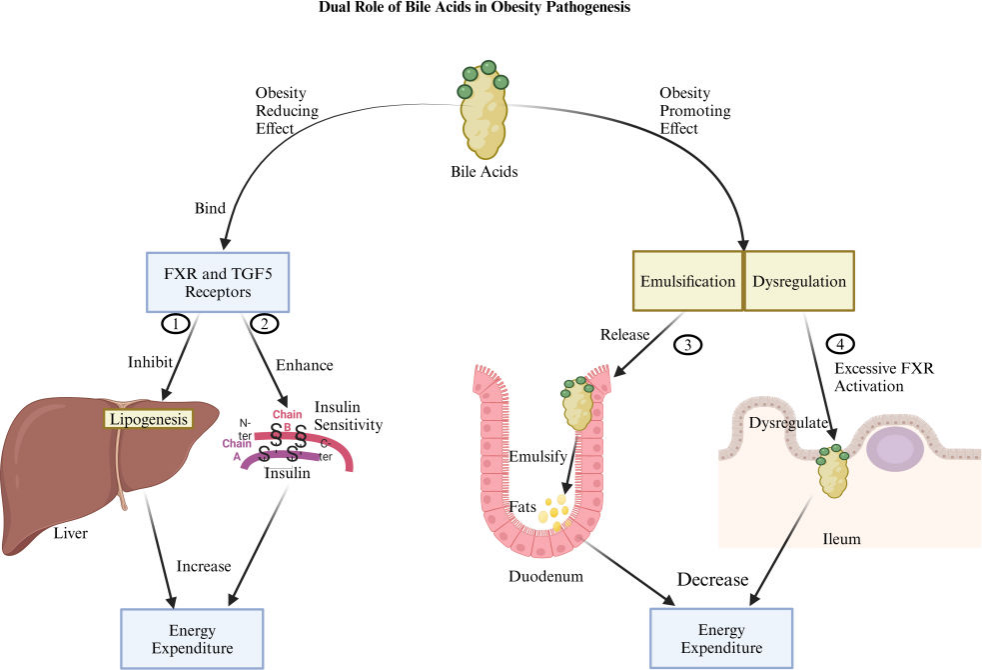
It illustrates the dual role of bile acids in obesity pathogenesis is as follows: (1) Bile acids demonstrate an obesity-reducing effect by binding to FXR and TGR5 receptors, which, when activated, inhibit lipogenesis in the liver and increase energy expenditure. (2) Similarly, the activation of these receptors enhances insulin sensitivity and increases energy expenditure. (3) Conversely, bile acids also promote obesity by emulsifying dietary fats in the duodenum, leading to increased fat storage and a subsequent decrease in energy expenditure. (4) Additionally, bile acids promote obesity due to dysregulation in the ileum caused by excessive FXR activation, which also leads to a decrease in energy expenditure. Created with BioRender.com.

TGR5, highly expressed in BAT and other metabolic tissues, also plays a significant role in obesity management. Activation of TGR5 stimulates pathways linked to lipid and carbohydrate metabolism, energy expenditure, and inflammation (81). Studies in Tgr5^+/+^ mice have shown that the bile acid mimetic INT-777 activates TGR5, leading to increased mitochondrial biogenesis, enhanced mitochondrial β-oxidation, and improved mitochondrial function (83). A pilot study in 12 healthy women revealed that CDCA supplementation increased BAT activity and whole-body energy expenditure (84). Similarly, research on primary human brown adipocytes showed that CDCA and TGR5 agonists promoted mitochondrial uncoupling, an effect not observed in white adipocytes, indicating TGR5’s role in energy expenditure (84). Additionally, TGR5 signaling in enteroendocrine L cells induces the release of gastrointestinal hormones, including PYY and GLP1. These hormones regulate appetite and energy balance by promoting satiety, underscoring TGR5’s function in bridging bile acid signaling with metabolic homeostasis (85). However, bile acids can also promote obesity under certain conditions, especially through lipid emulsification and absorption (Fig. 3). Increased bile acid production and enhanced enterohepatic circulation can elevate dietary fat absorption, leading to adipose tissue expansion in the context of caloric excess (86). Dysregulated bile acid signaling, particularly excessive FXR activation in the intestine, has been associated with reduced energy expenditure and increased lipid storage, promoting obesity in certain pathological states (87).

Interactions between bile acids and gut microbiota further influence their impact on obesity. Some bacteria, such as *E. cloacae* and *C. aerofaciens*, modulate bile acid metabolism to enhance lipid absorption, potentially increasing adiposity (7, 26, 86). In contrast, beneficial bacteria like *A. muciniphila* activate FXR and TGR5, improving lipid and glucose metabolism and promoting energy expenditure (88). *L. rhamnosus*, *L. gasseri*, *B. longum*, and *B. animalis* contribute to a beneficial bile acid profile that supports metabolic health by enhancing insulin sensitivity and reducing adiposity (13–16). Similarly, EcN-GM, a probiotic, can be engineered to produce secondary bile acids, which may influence metabolic balance (17).

## ENDOCANNABINOIDS (eCBs)

**Fig 4:**
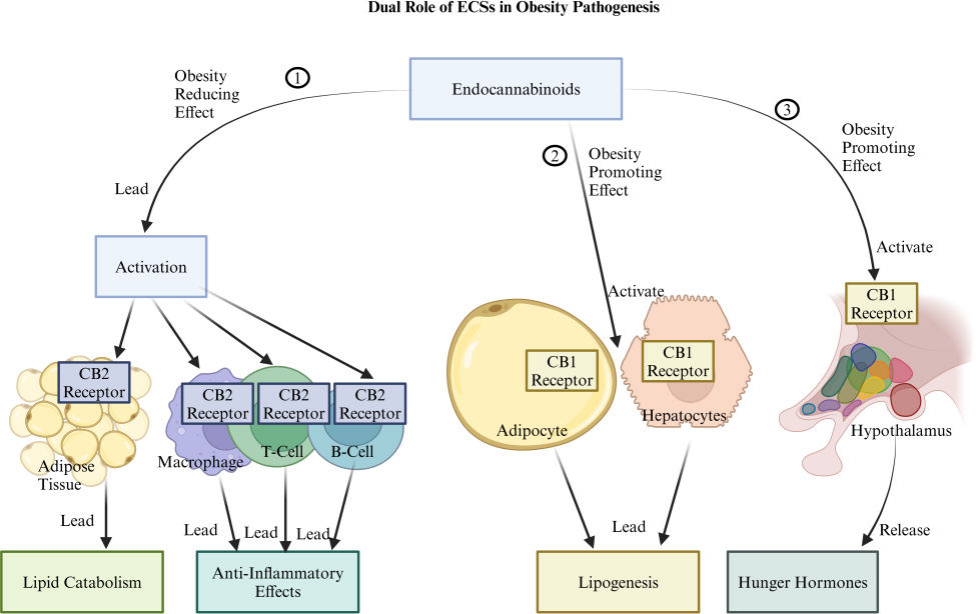
The figure illustrates the dual role of the endocannabinoid system (ECS) in obesity pathogenesis is as follows: (1) It demonstrates an obesity-reducing effect where ECS leads to the activation of CB2 receptors in adipose tissues and immune cells (macrophages, T cells, and B cells). This activation results in lipid catabolism in adipose tissue and anti-inflammatory effects from these immune cells, both collectively reducing obesity. (2) It demonstrates an obesity-promoting effect where ECS activates CB1 receptors in adipocytes and hepatocytes, leading to lipogenesis and promoting obesity. (3) It demonstrates an obesity-promoting effect where ECS activates CB1 receptors in the hypothalamus, resulting in the release of hunger hormones, increased appetite, and ultimately, obesity. Created with BioRender.com.

The eCB system (ECS) is integral to various physiological processes, including appetite regulation, glucose and lipid metabolism, immunity, and inflammation, and it plays a key role in mediating interactions between microbiota and host (89). Discovered in the late 20th century, the two primary cannabinoid receptors, CB1 and CB2, are activated by eCBs like anandamide (AEA) and 2-arachidonoylglycerol, which function as bioactive lipids affecting multiple systems, including adipose tissue metabolism (89). The ECS’s influence on energy balance is bidirectional, as it can promote both obesity and anti-obesity effects depending on the pathway involved (Fig. 4). CB1 receptors, highly expressed in brain regions such as the hypothalamus, are pivotal in driving hyperphagic behavior and promoting “hedonic eating”—the consumption of energy-dense, palatable foods (90, 91). This CB1 activation may foster energy storage and adiposity by enhancing lipogenesis in peripheral tissues like adipocytes and hepatocytes. Chronic CB1 activation in obesity may lead to insulin resistance, lipid accumulation, and metabolic dysfunction (92). Conversely, CB2 receptors, primarily found in immune cells and peripheral tissues, counteract the effects of CB1 by promoting anti-inflammatory responses, enhancing lipid catabolism, and increasing energy expenditure (93).

The ECS’s regulation of metabolic health also involves interactions with gut microbiota. Certain bacteria, such as *E. cloacae* and *C. aerofaciens*, have been associated with metabolic dysregulation, potentially activating CB1 receptors to increase lipogenesis and insulin resistance, thereby promoting obesity (26). On the other hand, beneficial bacteria like *A. muciniphila*, *L. rhamnosus*, *L. gasseri*, *B. longum*, and *E. coli* Nissle 1917 appear to influence CB2 receptor pathways, leading to reduced inflammation, enhanced lipid catabolism, and improved energy expenditure, thus supporting metabolic health and countering obesity (13–17). In obesity, elevated levels of AEA have been shown to increase gut permeability through CB1-dependent mechanisms, allowing translocation of LPS into circulation. This process, known as metabolic endotoxemia, perpetuates a cycle of gut barrier dysfunction, elevated LPS, and adipose tissue dysregulation (37, 94). Experimental activation of the ECS in animal studies has led to increased adipogenesis and impaired gut barrier function, emphasizing the interplay between the ECS, gut microbiota, and adipose tissue (94, 95).

Alterations in the ECS tone—evidenced by changes in eCB levels, receptor expression, and enzyme activity—are often observed in obesity and are linked to dysbiosis and metabolic imbalances (37). Studies with genetically obese (ob/ob) and diabetic (db/db) mice, as well as mice with diet-induced obesity, demonstrate that shifts in gut microbiota composition coincide with altered eCB signaling, further supporting the connection between bioactive lipids, gut microbiota, and adipose tissue metabolism (41, 96). Research into enzymes like N-acylphosphatidylethanolamine-hydrolysing phospholipase D (NAPEPLD), essential for synthesizing eCB bioactive lipids, has deepened our understanding of the ECS’s role in metabolic regulation (97). Mice with adipocyte-specific deletion of NAPEPLD spontaneously develop obesity, insulin resistance, and inflammation, even on a standard diet (97). These mice also show reduced thermogenic activity in adipose tissue and notable alterations in gut microbiota composition. When the altered microbiota from these mice was transferred to germ-free mice, it reproduced the obesity phenotype, implicating gut microbiota as a causal factor in the observed metabolic effects (97). This bidirectional communication between the ECS and gut microbiota highlights the potential of microbiota to influence host eCB signaling. Bioinformatics analyses have found that gut microbiota can produce N-acyl amides structurally similar to human GPCR ligands (98). Studies in gnotobiotic mice colonized with bacteria capable of producing N-acyl serinols revealed reduced blood glucose levels, likely through interaction with host GPR119, suggesting that microbiota may directly affect host GPCR pathways (98).

## OXYLIPINS

**Fig 5:**
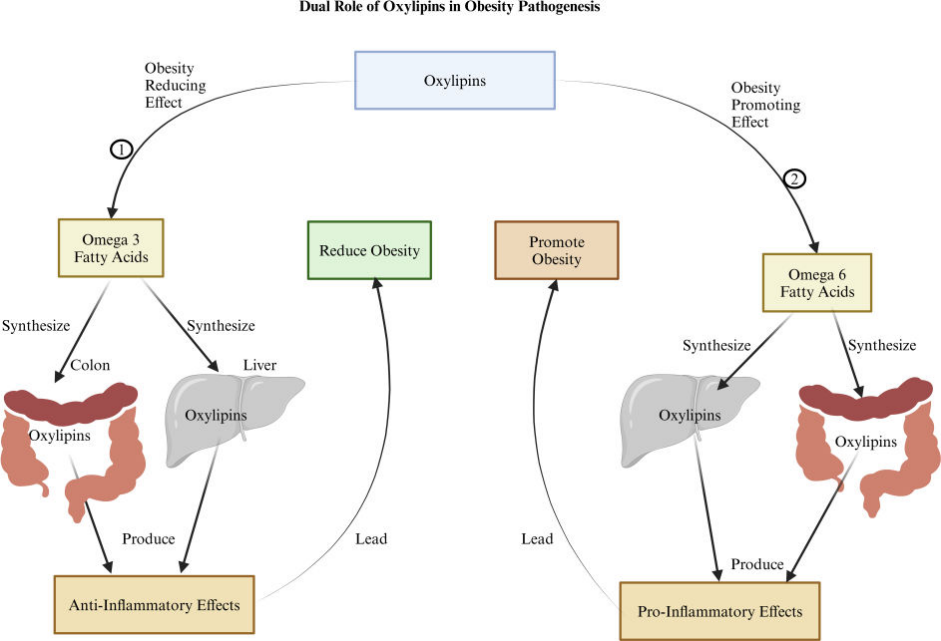
The figure illustrates the dual role of oxylipins in obesity pathogenesis is as follows: (1) It demonstrates an obesity-reducing effect where oxylipins are synthesized in the colon and liver from omega-3 fatty acids. These oxylipins (such as resolvins, protectins, and maresins) are typically anti-inflammatory and contribute to reducing obesity by improving metabolic health and decreasing inflammation. (2) Conversely, it demonstrates an obesity-promoting effect where oxylipins are synthesized in the colon and liver from omega-6 fatty acids. These oxylipins (such as prostaglandins and leukotrienes) are generally pro-inflammatory and contribute to promoting obesity by increasing inflammation, which is linked to the development of obesity. Created with BioRender.com.

Oxylipins, bioactive lipid mediators derived from the enzymatic oxidation of polyunsaturated fatty acids, play critical roles in regulating inflammation, immune responses, and various physiological functions (99). Their influence on obesity is complex, with pro- and anti-inflammatory oxylipins impacting metabolic and immune pathways differently (Fig. 5). Pro-inflammatory oxylipins, primarily synthesized from omega-6 fatty acids like arachidonic acid, produce mediators such as prostaglandins and leukotrienes, which drive chronic low-grade inflammation (99). This inflammation is a key pathophysiological factor in obesity, insulin resistance, and metabolic syndrome. Conversely, oxylipins derived from omega-3 fatty acids, including resolvins, protectins, and maresins, possess anti-inflammatory properties that can help counteract inflammation, potentially alleviating metabolic disturbances associated with obesity (100). The gut microbiota significantly influences oxylipin synthesis and metabolism. Dysbiosis, often present in obesity, skews oxylipin production toward a pro-inflammatory profile, exacerbating adiposity and related metabolic issues (2). Targeting microbial homeostasis through therapeutic strategies may shift oxylipin synthesis toward anti-inflammatory profiles, thus reducing inflammation and supporting obesity management (99). Certain gut bacteria may influence oxylipin profiles through either pro-inflammatory or anti-inflammatory pathways. For instance, *E. cloacae* and *C. aerofaciens* may affect metabolic dysregulation and promote pro-inflammatory oxylipins, exacerbating adiposity and comorbidities (101, 102). In contrast, beneficial bacteria such as *A. muciniphila* may support anti-inflammatory oxylipin production, promoting metabolic health and reducing obesity risk (103). *L. rhamnosus* and *L. gasseri* are linked to anti-inflammatory oxylipin synthesis, supporting metabolic balance, while *B. longum* and *B. animalis* also promote a balanced metabolic state, lowering obesity risk (104, 105).

Among oxylipins, 12,13-diHOME (isoleukotoxin diol), formed from linoleic acid via cytochrome P450 and soluble epoxide hydrolase, has gained attention for its roles in adipose tissue regulation (106). Primarily produced by brown and beige adipose tissue, 12,13-diHOME levels are modulated by factors like exercise, diet, and temperature. In obese adolescent males, 12,13-diHOME concentrations were found to be lower than in their normal-weight peers, although levels increased with acute exercise (107). In mice with high-fat diet-induced obesity, administering 12,13-diHOME promoted fatty acid transport into BAT, reduced circulating triglyceride levels, and increased LPL gene expression, facilitating triglyceride hydrolysis (108). Interestingly, some gut bacteria also produce 12,13-diHOME. For instance, *Dysosmobacter welbionis* has been identified as a producer of this oxylipin. In mouse studies, administering *D. welbionis* significantly reduced BAT whitening (a marker of dysfunction) induced by a high-fat diet and increased mitochondrial activity in BAT, highlighting the microbiota’s potential role in modulating oxylipin profiles to improve metabolic health (109, 110).

## SUCCINATE AND GPR91

**Fig 6:**
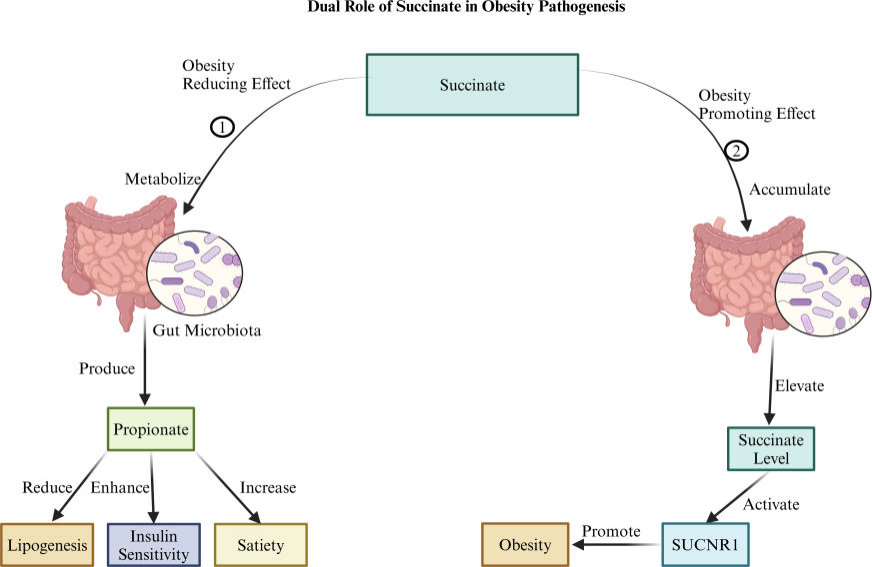
The figure illustrates the dual role of succinate in obesity pathogenesis is as follows: (1) It demonstrates an obesity-reducing effect where succinate is metabolized by the gut microbiota in the colon, leading to the production of propionate, which reduces lipogenesis, enhances insulin sensitivity, and increases satiety, collectively reducing obesity. (2) It demonstrates an obesity-promoting effect where succinate is not metabolized, leading to its accumulation. This elevated succinate level can activate succinate receptor 1 (SUCNR1), promoting inflammation and metabolic dysregulation, thereby contributing to obesity. Created with BioRender.com.

Succinate, a crucial intermediate in the tricarboxylic acid cycle, functions as a significant signaling molecule within the gut microbiota, with context-dependent effects on obesity (Fig. 6). Elevated succinate levels are associated with obesity pathogenesis primarily through the activation of succinate receptor 1 (SUCNR1 or GPR91). This receptor, widely expressed in tissues such as the kidney, liver, heart, retina, and adipose tissue, facilitates succinate’s pro-inflammatory role by promoting the release of cytokines, contributing to chronic low-grade inflammation—a core mechanism in obesity and insulin resistance (111). Additionally, succinate may shift energy metabolism towards glycolysis, thereby promoting energy imbalance and adiposity (112). While succinate is central to cellular metabolism, it can also be generated through microbial carbohydrate fermentation (111, 112). Certain gut bacteria utilize the succinate pathway to produce propionate, an SCFA with established metabolic benefits, including reduced lipogenesis, enhanced insulin sensitivity, and increased satiety. This conversion underscores succinate’s dual potential: under specific microbial environments, it may foster anti-obesity effects through propionate production (113). The effects of succinate on obesity thus depend on factors like concentration, the surrounding microbial environment, and the metabolic pathways activated (111–113). For instance, *E. cloacae* and *C. aerofaciens* may elevate succinate levels, activating SUCNR1 and fostering inflammation, which disrupts metabolic homeostasis and exacerbates obesity (26). Interestingly, inverse correlations exist between the abundance of *A. muciniphila*, which produces succinate during mucin degradation (114), and obesity, diabetes, and related metabolic disorders (115). Additionally, *A. muciniphila* may support succinate conversion to propionate through interactions with other gut bacteria. The introduction of succinate producers like *P. distasonis* has also been shown to ameliorate metabolic dysfunctions in mice (116). Other beneficial microbes, including *L. rhamnosus*, *L. gasseri*, *B. longum*, *B. animalis*, and *E. coli* Nissle 1917, may also promote favorable succinate metabolism, supporting metabolic health (13–17).

Recent studies in mice link dietary fiber consumption with increased succinate production, particularly from *Prevotella* species, illustrating succinate’s role as a beneficial microbial metabolite tied to dietary intake (117, 118). In rodent models, elevated succinate levels correlate with obesity, type 2 diabetes, and hypertension, whereas human studies do not find a similar association, suggesting species-specific differences in succinate’s role in metabolic health (119). In mouse studies, the deletion of SUCNR1 (SUCNR1^−/−^) revealed its complex role in regulating adipose tissue and glucose homeostasis (119, 120). SUCNR1 knockout mice exhibited reduced adipocyte size, increased energy expenditure, and improved glucose regulation, yet long-term high-fat diets led to increased adiposity, hyperglycemia, and liver damage, emphasizing SUCNR1’s nuanced role in energy sensing and obesity (119, 120). Beyond metabolic disorders, succinate is studied in inflammatory conditions like Crohn’s disease (121). Elevated plasma succinate and SUCNR1 expression in adipose tissues and macrophages have been observed in individuals with active Crohn’s. Interestingly, succinate’s role in inflammation may extend to its impact on adipose tissue. Treatment with succinate in adipose-derived stem cells has been shown to increase markers of beige adipose tissue, suggesting that succinate could potentially contribute to converting white to beige adipocytes under inflammatory conditions. However, its relevance to obesity and the broader implications for metabolic health remain to be fully explored (121).

## FIAF

**Fig 7:**
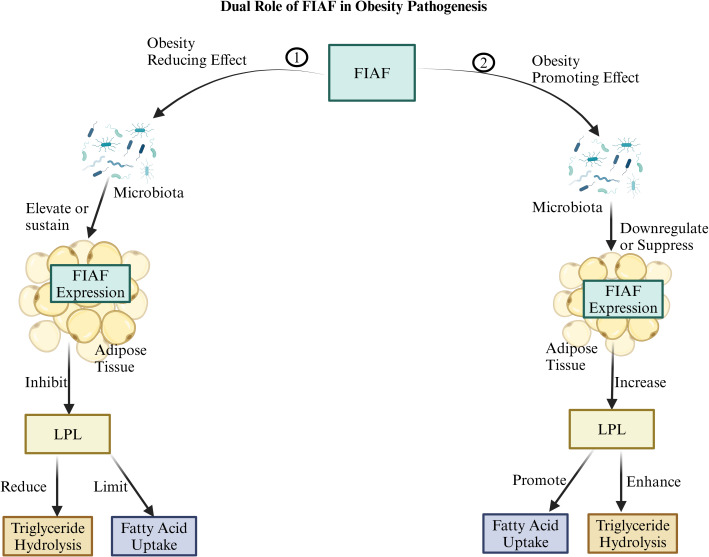
The figure illustrates the dual role of fasting-induced adipose factor (FIAF), also known as ANGPTL4, in obesity pathogenesis is as follows: (1) It demonstrates an obesity-reducing effect where FIAF expression is elevated or sustained in adipose tissue, potentially due to the influence of gut microbiota. This elevated or sustained FIAF level inhibits lipoprotein lipase (LPL), reducing triglyceride hydrolysis and limiting fatty acid uptake into adipose tissue. (2) It demonstrates an obesity-promoting effect where FIAF expression is downregulated or suppressed in adipose tissue, potentially due to gut microbiota influence. This downregulation or suppression of FIAF levels increases LPL activity, which enhances triglyceride hydrolysis and promotes fatty acid uptake into adipose tissue. Created with BioRender.com.

FIAF, also known as angiopoietin-like protein 4 (ANGPTL4), is a circulating protein produced by various tissues, including the intestine, liver, and adipose tissue, particularly in response to fasting (122). It is regulated by peroxisome proliferator-activated receptor (PPAR) proteins and plays a critical role in lipid metabolism (123). FIAF functions by inhibiting lipoprotein lipase (LPL), an enzyme essential for triglyceride hydrolysis in circulating lipoproteins. This inhibition decreases fatty acid uptake into adipose and muscle tissues, potentially preventing excessive fat storage and offering a mechanism to mitigate obesity (124). However, FIAF’s role in obesity is complex, as it is influenced by its expression levels, gut microbiota interactions, and broader metabolic context (Fig. 7). The gut microbiota significantly modulates FIAF expression, with certain microbial populations either upregulating or downregulating FIAF, thus affecting lipid storage (125). For instance, *E. cloacae* and *C. aerofaciens* have been associated with reduced FIAF levels, promoting LPL activity, fatty acid uptake, and adiposity (124, 125). Dysbiosis, or microbial imbalance, may further disrupt FIAF expression, potentially increasing obesity risk in specific metabolic environments (2, 126). Conversely, beneficial bacteria such as *A. muciniphila can* upregulate FIAF expression, limiting LPL activity, reducing fat storage, and promoting a leaner phenotype. Similarly, *L. rhamnosus*, *L. gasseri*, *B. longum*, *B. animalis*, and *E. coli* Nissle 1917 may contribute to FIAF regulation, fostering reduced fat accumulation and metabolic balance (13–17).

Research in germ-free mice has provided insights into FIAF’s role in metabolism. FIAF is constitutively expressed in germ-free mice, and colonization with gut microbiota reduces FIAF expression, thereby increasing LPL activity and body fat mass (127, 128). Interestingly, germ-free mice with FIAF gene knockouts lose their resistance to high-fat diet-induced obesity (127, 128). However, the association between gut microbiota and obesity remains inconclusive, with studies yielding mixed results regarding microbiota’s protective effect against obesity. Replication attempts of initial findings have sometimes failed, indicating that the relationship between gut bacteria and metabolic disease is complex and warrants further investigation. Notably, while high-fat diets in germ-free mice elevated FIAF expression in the intestine, this effect was not observed in circulating FIAF levels (129). The mechanism by which the gut microbiota regulates FIAF remains only partially understood. Some studies indicate that specific bacteria can enhance FIAF expression in human intestinal cells and increase circulating levels in mice, suggesting that microbiota modulation could influence FIAF (129). Additionally, FIAF may play a role in central energy metabolism regulation via hypothalamic AMPK inhibition, although it is unclear whether gut microbiota directly impacts hypothalamic FIAF (130). Overall, these findings underscore the intricate interactions between gut microbiota, FIAF, and metabolic regulation, highlighting FIAF’s potential as a therapeutic target in obesity and metabolic diseases.

## MICROBIOTA-DRIVEN THERAPEUTICS FOR OBESITY MANAGEMENT

A microbiota-targeted therapeutic approach to combat obesity involves modifying metabolites and regulating adipose tissue metabolism through specific dietary and bacterial interventions (130). Key dietary components such as resveratrol, capsaicin, quercetin, epigallocatechin-3-gallate, berberine, rhubarb extract, and camu camu have been shown to promote the beiging or browning of adipose tissue, activating markers such as uncoupling protein 1 (UCP1), DIO2, CPT1α, Cidea, PGC1α, SIRT1, and BMP7 (131–141). These compounds support fat oxidation, cold-induced thermogenesis, and mitochondrial function, thus protecting against diet-induced obesity in animal models. A microbiota-centric approach further enhances this effect by promoting beneficial bacteria like *A. muciniphila* and *D. welbionis*, which increase BAT activity, enhance fatty acid oxidation, and improve gut barrier function (110, 142). The production of bioactive lipids, such as 12,13-diHOME, by *D. welbionis* has been shown to decrease BAT whitening and boost mitochondrial activity, demonstrating the potential of targeted microbiota modulation in metabolic health. In the context of SCFAs, promoting beneficial SCFA production, particularly butyrate, by cultivating *A. muciniphila*, *Lactobacillus spp.*, and *Bifidobacterium* spp*.* enhances gut barrier integrity, reduces inflammation, and activates AMPK, promoting energy expenditure (143). Adjusting SCFA metabolism to favor butyrate over acetate may reduce hepatic lipogenesis, while inhibiting obesogenic bacteria like *E. cloacae* and *C. aerofaciens*, which enhance energy harvest, supports a leaner phenotype (144). For bile acid modulation, fostering beneficial bacteria like *A. muciniphila*, *L. rhamnosus*, and *Bifidobacterium* spp. can enhance bile acid profiles that activate FXR and TGR5, improving lipid and glucose metabolism, increasing energy expenditure, and reducing inflammation (Tables 1 and 2). Zheng et al. demonstrated that inhibiting bile acid biosynthesis under high-fat diet conditions mitigates gut microbiome alterations and improves obesity phenotypes by targeting bile acid pools or suppressing the microbiota, highlighting the impact of bile acids on microbiota composition and their potential for reducing obesity-related traits (145).

A balanced ECS approach may involve inhibiting CB1 to reduce hyperphagia, lipogenesis, and insulin resistance while activating CB2 to encourage anti-inflammatory responses and lipid breakdown (Tables 1 and 2). Microbiota species like *A. muciniphila*, *L. rhamnosus*, and *Bifidobacterium* spp. can support CB2 pathway activation, fostering metabolic health and reducing inflammation, which aids in obesity reduction. In oxylipin modulation, beneficial bacteria like *A. muciniphila* and *Lactobacillus* spp. can shift oxylipin production toward anti-inflammatory mediators, such as resolvins and protectins derived from omega-3 fatty acids, which reduce obesity risk by mitigating inflammation (Tables 1 and 2). Conversely, inhibiting bacteria like *E. cloacae* and *C. aerofaciens*, which contribute to pro-inflammatory oxylipins, can help curb obesity progression. For succinate modulation, promoting bacteria that convert succinate into propionate—such as *A. muciniphila* and *Bifidobacterium* spp.—enhances anti-obesity effects by reducing lipogenesis, improving insulin sensitivity, and promoting satiety (Tables 1 and 2). Inhibiting pro-inflammatory succinate pathways by reducing bacteria like *E. cloacae* and *C. aerofaciens* can prevent metabolic dysregulation, allowing succinate’s anti-obesogenic potential to be maximized. Finally, modulation of FIAF can further support obesity management by promoting bacteria like *A. muciniphila*, *Lactobacillus* spp., and *Bifidobacterium* spp., which upregulate FIAF to inhibit LPL activity and reduce fat storage, fostering a leaner body composition (Tables 1 and 2). Inhibiting bacteria that downregulate FIAF, such as *E. cloacae*, can further support reduced adiposity and enhance overall metabolic health. Together, these strategies highlight the potential of targeted, microbiota-based interventions that focus on specific metabolic pathways, offering a possible personalized approach for managing obesity and improving metabolic health.

Although probiotic and microbiota transplantation therapies are showing promise in managing obesity and metabolic disorders, several challenges remain that must be addressed to enhance their efficacy and consistency. A key challenge is the variability in patient outcomes, as even with similar interventions like probiotics or microbiota transplants, patients often experience different results, influenced by factors such as baseline microbiota composition, which affects the effectiveness of the treatment. Additionally, host genetics can play a role, as genetic differences may influence immune responses, nutrient metabolism, and microbiota interactions, affecting therapeutic success. Understanding these factors can help tailor interventions for better outcomes. Another limitation is the need for mechanistic insights, as while the benefits of microbiota interventions are recognized, there is a significant gap in understanding the precise molecular mechanisms, and identifying how microbial metabolites like SCFAs or bile acids interact with host metabolic pathways is crucial for refining therapeutic targeting. Furthermore, integrating multi-omics approaches such as metagenomics, metabolomics, and proteomics can provide deeper insights into the complex interactions between the microbiota, immune system, and host metabolism. Finally, a conceptual model that integrates microbial metabolites, immune signaling, and systemic metabolism could optimize microbiota-based therapies for improved metabolic health.

## GUT–ADIPOSE AXIS AND METABOLIC HEALTH

Recent studies suggest that adipose tissue contains a distinct microbiota signature influenced by the host’s metabolic burden, providing new insights into metabolic health (2). However, studying this microbiota presents several challenges. Common detection methods, such as 16S rRNA gene sequencing, can be prone to contamination, limited sensitivity, and the inability to distinguish between viable and dead bacteria or reveal microbial function (146, 147). Complementary approaches like metagenomics, metatranscriptomics, and metabolomics are essential for deeper insights into the functional role of the adipose microbiota (146, 147). The translocation of microbes from the gut to adipose tissue remains unclear, but it may involve increased intestinal permeability, immune cell transport, or circulation through the portal vein or lymphatic system, particularly in metabolic disorders like obesity and type 2 diabetes (148). Studies showed that microbial signatures vary across different fat depots, such as subcutaneous and visceral fat, with individuals experiencing obesity often exhibiting higher bacterial loads but lower microbial diversity, which correlates with disrupted lipid metabolism and inflammation (147). An emerging area of interest is the role of microbiota in breast tissue and its association with the development of “pink” adipocytes during pregnancy and lactation (149). These specialized cells, which transdifferentiate from white adipocytes, produce milk and contribute to mammary gland function. Alterations in breast tissue microbiota may influence mammary health, adipocyte function, and even the progression of breast cancer (150). Understanding these connections could open avenues for new therapeutic strategies targeting the interplay between breast microbiota and adipose tissue.

The interaction between gut microbiota and adipose tissue, often referred to as the gut–adipose axis, represents a complex bidirectional communication network. This axis involves the exchange of signaling molecules, metabolites, and immune mediators between the gut and adipose tissue. Adipose tissue, once viewed merely as an energy reservoir, is now recognized as an active endocrine organ that secretes adipokines, cytokines, and other molecules with systemic effects (151). Simultaneously, the gut microbiota produces metabolites that regulate host metabolism and immune responses, emphasizing the dynamic interaction between these systems (152). Several biomarkers associated with obesity and insulin resistance have emerged from this interplay. Changes in microbial diversity and the abundance of specific taxa, such as *A. muciniphila*, are associated with improved metabolic health (153). Higher levels of *A. muciniphila* correlate with smaller adipocyte size and improved insulin sensitivity, although individual variability poses challenges in establishing definitive microbial signatures for metabolic dysfunction (153, 154). Microbial metabolites, such as SCFAs, secondary bile acids, and Trimethylamine-N-oxide (TMAO), also offer predictive value. Elevated SCFA levels are associated with reduced body weight, fat mass, glucose levels, and inflammation, while secondary bile acids improve BMI and insulin sensitivity (155). In contrast, increased TMAO levels correlate with higher BMI, insulin resistance, and oxidative stress (64). Additionally, circulating adipokines and inflammatory markers provide further insight into the relationship between adipose tissue health and metabolic outcomes. Individual variations in microbial responses to diet highlight the potential for personalized nutrition to mitigate metabolic risks (156). Genetic variations that affect host–microbe interactions may offer promising markers for assessing susceptibility to metabolic disorders (131). This intricate communication between the gut microbiota and adipose tissue offers exciting opportunities for identifying biomarkers that could help detect individuals at risk for obesity and insulin resistance. These biomarkers could guide early interventions and personalized strategies to improve metabolic outcomes. However, further longitudinal and mechanistic research is necessary to unravel the molecular mechanisms driving these interactions and validate the clinical utility of these biomarkers, paving the way for novel therapies targeting the gut–adipose axis (Table 3).

## TRANSLATING GUT MICROBIOME INTO METABOLIC THERAPIES

Although significant progress has been made in understanding the interactions between gut microbiota and adipose tissue, translating findings from *in vitro* and animal studies to humans remains challenging. Animal models like germ-free mice provide insights into specific microbial roles, but their altered metabolism and impaired immune systems limit their applicability to human physiology. Genetically obese mice, such as *ob/ob* and *db/db* strains, have advanced our understanding of obesity’s pathophysiology, but their reliance on leptin-related mutations—rare in humans—limits their relevance (157). Similarly, high-fat diet-induced obesity models capture some features of human obesity but fail to reflect the multifactorial influences of genetics, lifestyle, and environmental factors (158). The biological differences in genetics, diet, and microbiota composition between species further complicate the translation of animal findings to human populations, necessitating careful interpretation. The human gut microbiome is dynamic and highly individualized, shaped by factors such as diet, medications, stress, and lifestyle. This inter-individual variability makes it difficult to design standardized interventions with consistent outcomes across diverse populations (159). Changes in the microbiota can take time to manifest or fade over time, requiring sustained and iterative intervention strategies (160). Personalized treatments, tailored to individual microbiomes, genetic predispositions, and metabolic profiles, will be crucial to avoid unintended consequences, such as inflammation or disrupted energy balance. Furthermore, the pleiotropic effects of metabolites like SCFAs and oxylipins, which can exhibit both beneficial and harmful effects depending on the context, demand a nuanced, context-specific therapeutic approach.

As research advances, future therapeutic strategies will likely focus on precision microbiome modulation to optimize metabolic homeostasis (Tables 1 to 3). Targeting microbial consortia that regulate key metabolic pathways, such as SCFA biosynthesis and FIAF, offers promising potential. Promoting beneficial taxa like *A. muciniphila*, *Lactobacillus*, and *Bifidobacterium* may enhance anti-inflammatory responses, strengthen gut barrier integrity, and increase energy expenditure, helping mitigate adiposity. Additionally, modulating bile acid metabolism and the ECS through microbiome-based interventions could improve insulin sensitivity and reduce obesity risk. However, fine-tuning FIAF’s dual role in lipid metabolism—where inhibition of LPL may restrict free fatty acid availability—requires careful navigation. Clinical microbiome interventions face variability in the types of probiotics, dosages, and treatment durations used, complicating comparisons between studies. Optimal treatment regimens, including dosage, duration, and route of administration, are not well-defined, and inconsistent protocols contribute to mixed outcomes. Differences in metadata collection, including diet, lifestyle factors, and medication use, also hinder the ability to control for confounding factors and compare results across studies.

Most studies rely on fecal samples due to their non-invasive nature and sufficient biomass for analysis. However, fecal microbiota may not accurately reflect microbial communities throughout the gastrointestinal tract. Microbiota composition varies by region due to differences in nutrient availability, oxygen levels, and environmental conditions (161). The mucosal layer, where host–microbe interactions occur, often harbors distinct microbial communities from those found in the intestinal lumen. These differences underscore the need for more comprehensive sampling strategies beyond fecal analysis to fully understand microbiota’s role in metabolism and immune function. Gut microbiota profiling also presents challenges due to the absence of standardized workflows. Techniques such as 16S rRNA gene sequencing, shotgun metagenomics, and metatranscriptomics offer distinct insights but come with limitations (162). 16S rRNA sequencing identifies microbial composition but lacks functional resolution, while shotgun metagenomics captures functional genes and pathways but cannot reveal real-time gene expression. The high cost and complexity of interpreting metagenomic data, along with the lack of standardized bioinformatics pipelines, further complicate the comparison of results across studies (98). To unlock the therapeutic potential of the gut microbiota, future research must focus on refining sampling techniques, developing standardized protocols, and improving data analysis tools to enhance the comparability of studies. Establishing reliable biomarkers for tracking gut permeability and microbial translocation dynamics will also be essential, as current clinical markers remain insufficient. Personalized interventions tailored to individual microbiome compositions and metabolic profiles will be crucial in optimizing outcomes. Additionally, fine-tuning SCFA and oxylipin pathways to promote anti-obesogenic effects while preserving other physiological functions will be necessary for safe and effective interventions. Precision modulation of microbial pathways, including FIAF’s role in lipid metabolism, holds promise, though careful balancing will be required to avoid unintended disruptions in metabolic regulation.

## CONCLUSION

The interplay between gut microbiota, PAMPs, and obesity highlights the potential of precision microbiota modulation for obesity management. Key strategies may include enhancing beneficial taxa like *A. muciniphila*, *Lactobacillus*, and *Bifidobacterium* to support SCFA production and regulate FIAF for metabolic balance. However, individual microbiome variability, complex microbial metabolite roles, and ethical concerns can pose challenges. Establishing causal links between microbiota Advances in omics technologies are driving personalized medicine by enabling tailored treatments based on individual microbiome profiles, marking a critical shift toward more targeted and sustainable healthcare solutions.

## Data Availability

All data included in this study are available upon request from the corresponding author.
